# FTY720 inhibits mesothelioma growth in vitro and in a syngeneic mouse model

**DOI:** 10.1186/s12967-017-1158-z

**Published:** 2017-03-15

**Authors:** Agata Szymiczek, Sandra Pastorino, David Larson, Mika Tanji, Laura Pellegrini, Jiaming Xue, Shuangjing Li, Carlotta Giorgi, Paolo Pinton, Yasutaka Takinishi, Harvey I. Pass, Hideki Furuya, Giovanni Gaudino, Andrea Napolitano, Michele Carbone, Haining Yang

**Affiliations:** 1 Thoracic Oncology Program, University of Hawaii Cancer Center, 701 Ilalo Street, Honolulu, HI 96813 USA; 2 Department of Morphology-Surgery-Experimental Medicine, University of Ferrara, Ferrara, Italy; 3 Department of Cardiothoracic Surgery, New York University Langone Medical Center, New York, NY 10065 USA

**Keywords:** Malignant mesothelioma, Therapeutic, Drug repurposing, FTY720, PP2A, Apoptosis

## Abstract

**Background:**

Malignant mesothelioma (MM) is a very aggressive type of cancer, with a dismal prognosis and inherent resistance to chemotherapeutics. Development and evaluation of new therapeutic approaches is highly needed. Immunosuppressant FTY720, approved for multiple sclerosis treatment, has recently raised attention for its anti-tumor activity in a variety of cancers. However, its therapeutic potential in MM has not been evaluated yet.

**Methods:**

Cell viability and anchorage–independent growth were evaluated in a panel of MM cell lines and human mesothelial cells (HM) upon FTY720 treatment to assess in vitro anti-tumor efficacy. The mechanism of action of FTY720 in MM was assessed by measuring the activity of phosphatase protein 2A (PP2A)—a major target of FTY720. The binding of the endogenous inhibitor SET to PP2A in presence of FTY720 was evaluated by immunoblotting and immunoprecipitation. Signaling and activation of programmed cell death were evaluated by immunoblotting and flow cytometry. A syngeneic mouse model was used to evaluate anti-tumor efficacy and toxicity profile of FTY720 in vivo.

**Results:**

We show that FTY720 significantly suppressed MM cell viability and anchorage–independent growth without affecting normal HM cells. FTY720 inhibited the phosphatase activity of PP2A by displacement of SET protein, which appeared overexpressed in MM, as compared to HM cells. FTY720 promoted AKT dephosphorylation and Bcl-2 degradation, leading to induction of programmed cell death, as demonstrated by caspase-3 and PARP activation, as well as by cytochrome c and AIF intracellular translocation. Moreover, FTY720 administration in vivo effectively reduced tumor burden in mice without apparent toxicity.

**Conclusions:**

Our preclinical data indicate that FTY720 is a potentially promising therapeutic agent for MM treatment.

**Electronic supplementary material:**

The online version of this article (doi:10.1186/s12967-017-1158-z) contains supplementary material, which is available to authorized users.

## Background

Malignant mesothelioma (MM) is a neoplasm arising from mesothelial cells lining the pleural, peritoneal, and pericardial cavities [1]. MM causes more than 40,000 deaths per year worldwide [1–3]. MM development is primarily associated with occupational and environmental exposure to carcinogenic mineral fibers, such as asbestos and erionite [1]. Asbestos deposition in the pleura promotes a chronic inflammation process that, over the years, leads to MM [4, 5] Despite the banning and/or severe restrictions implemented in the past decades, on the use of asbestos, the frequency of MM has remained stable for over a decade in the US, and it is still increasing in Europe, and in rapidly industrializing countries, such as India and China, that continue to use asbestos products [6–9]. The continuous rise in MM, occurring worldwide, may be due to several factors, including asbestos in place and the increased development of rural areas containing asbestos or other mineral fibers [10–12], as well as to other carcinogens that cause MM in animals, and to which humans have been exposed [13, 14]. Moreover, we discovered a novel hereditary cancer syndrome characterized by a very high risk of MM and other cancers, caused by mutations in the tumor suppressor gene BRCA1 associated protein-1 (BAP1) [15–17]. We found that even a minimal exposure to asbestos may induce MM in germline BAP1 heterozygous mice [18].

MM is a very aggressive cancer with poor prognosis and is refractory to most therapeutic modalities. Patients’ median survival is about 12–13 months from diagnosis [8] and is extended by only 11 weeks with the standard of care for pleural MM, based on the use of cisplatin/pemetrexed combination therapy [19]. Therefore, the development of new therapeutic approaches with unique mechanisms of action against the MM malignant phenotype is highly needed.

FTY720 (Fingolimod, Gilenya^®^) is an FDA-approved immunosuppressant currently used in the treatment of multiple sclerosis (MS) [20]. The immunosuppressant properties of FTY720 are mediated by its phosphorylated metabolite (FTY720-P), which, as analog of sphingosine-1 phosphate (S1P), modulates S1P receptor signaling leading to sequestration of lymphocytes in lymphoid tissues [21]. Over the last few years, several studies have shown that FTY720 was also effective on cancer-associated targets, both in vitro and in vivo, suggesting its use as an innovative anti-cancer agent [22]. Inhibition of tumor growth by FTY720 has been shown in vitro and in vivo in several cancer models including: uterine cervical cancer [23], hematologic malignancies [24], hepatocellular carcinoma [25], breast [26], lung [27], and prostate [28] cancers. FTY720 has been also shown to promote chemo-sensitization in hepatocellular and renal cell carcinomas [29, 30], suggesting its possible effectiveness in combination therapies. As opposed to its immunosuppressant activities, the anti-cancer properties of FTY720 are known to be mainly mediated by its non-phosphorylated form, via inhibition of sphingosine kinase 1 (SphK1) and activation of the protein phosphatase 2A (PP2A), leading to the reduction in cell survival [24, 31, 32]. FTY720 may also modulate motility and invasiveness of cancer cells, however whether this property is related to the phosphorylated or unphosphorylated form of FTY720 has not been addressed yet [26, 33, 34].

The anti-cancer properties of FTY720 have not been explored in relation to MM, however, its molecular targets, SphK1 and PP2A are known to be involved in MM cell proliferation and survival [35–37], prompting us to investigate the possible anti-cancer activity of FTY720 in MM cells and in a syngeneic MM mouse model.

## Methods

### Cell cultures and compounds

Human normal mesothelial cells (HM) were obtained from pleural effusions of non-cancer patients (cell identity was confirmed by immunohistochemistry using Wilms Tumor 1 (WT-1), calretinin and pancytokeratin as mesothelial markers [6], as well as by histopathology analysis by a board-certified pathologist, M.C.) and cultured as described [38]. Malignant MM cell lines PPM-MILL, PHI (originally denominated as HP3, [39]) and HMESO were established in tissue culture from surgically resected MM specimens as described [40]. REN MM cells were provided by Dr. Steven Albelda (University of Pennsylvania, Philadelphia, PA). NCI-H226 and NCI-H2052 and NCI-H2452 MM cells were obtained from American Type Culture Collection (ATCC, Manassas, VA, US). MM cells were routinely characterized and authenticated in our lab by immunostaining using antibodies against mesothelial markers (see above), and in collaboration with Genetica DNA Laboratories (Cincinnati, OH, US). Human umbilical vein endothelial cells (HUVEC) were purchased from Cell Applications, Inc (San Diego, CA, US). AB1 mouse MM cells (Balb/c) were kindly provided by Dr. Bruce W. S. Robinson [41]. All MM cells were cultured in D-MEM (Corning, NY, US) supplemented with 10% Fetal Bovine Serum (FBS, Gibco, ThermoFisher, Waltham, US), —hereinafter referred to as ‘complete medium’, unless otherwise specified. HM cells were cultured in D-MEM supplemented with 20% FBS, unless otherwise specified, HUVEC were cultured in F-12K Medium (ATCC, VA, US), supplemented with 0.04 mg/ml endothelial cell growth supplement (ECGS, Sigma-Aldrich, MO, US), 0.1 mg/ml heparin (Sigma-Aldrich, MO, US) and 10% FBS. FTY720 and FTY720-Phosphate (FTY720-P) were purchased from Cayman Chemical (MI, US). All stock solutions were prepared in DMSO.

### Cell viability and cell death assays

Cells (1 × 10^3^ per well) were grown in 96-well plates and treated in media supplemented with 1% FBS. Cell viability was assessed using the Alamar Blue assay (AbD Serotec, Raleigh, NC) according to the manufacturer’s instructions. Apoptosis was assessed as phosphatidylserine exposure, using the Annexin V-FITC apoptosis kit (BD Biosciences, San Jose, CA) followed by flow cytometric analysis (LSRFortessa, BD Biosciences).

### Migration assay

The in vitro cell migration assay was carried out using Costar Transwell® permeable polycarbonate supports (8.0-μm pores) in 24-well plates (Corning Inc., NY, US). Inserts were covered with 20 μg/ml fibronectin (Sigma-Aldrich). 1 × 10^5^ MILL and PHI cells were first treated with FTY720 (2 μM) or DMSO (0.003%) in serum-free DMEM for 3 h, in suspension. Then 200 μl of cell suspension was seeded in the inserts. The cells were allowed to migrate toward 2% FBS DMEM for 3 h Inserts were then collected, and the upper chamber was carefully cleaned to remove un-migrated cells. Migrate cells, laying on the lower surface of the insert, were then stained using HEMA 3 staining kit (Millipore, MA, US). The migrated cells were visualized under light microscope and counted using the ImageJ software, from three images taken from each well. Experiment was done in duplicate and performed two times.

### Soft agar assay

Anchorage-independent soft agar growth experiments were carried out in triplicates, in 6-well plates. The base agar layer contained 0.8% agar in complete culture medium, the upper layer contained 4 × 10^3^ cells per well seeded in 0.4% agar, dissolved in complete culture medium. Two millilitre of complete medium containing vehicle (0.01% DMSO) or 2 μM FTY720 treatment were added on the top of each well and were replaced every 3 days. The number and size of colonies were determined after 4–6 weeks of culture. All colonies larger than 0.1 mm in diameter were counted using the ImageJ software (NIH, Bethesda, MD).

### PP2A enzymatic activity assay

PP2A activity was measured using the PP2A immunoprecipitation phosphatase assay kit (Millipore, CA), according to the manufacturer’s instructions. In brief, the cells were harvested in lysis buffer (50 mM Tris HCl, pH 7, 2 mM EDTA, 1% Nonidet P-40, 1 µM PMSF, and protease inhibitors). Total protein concentration was determined using BCA Assay (Pierce Biotechnology, ThermoFisher). Lysates (200 µg) were incubated with 1 µg/ml anti-PP2A (C subunit, clone 1D6), or with IgG2bk as control, followed by incubation with protein A agarose at 4 °C for 2 h with constant rocking. The immunoprecipitates were washed 3 times with washing buffer and incubated with the PP2A substrate threonine phosphopeptide (final concentration 750 μM) for 10 min at 30 °C in a shaking incubator. At the completion of incubation, 25 μl of the reaction mixture were transferred to 96-well microtiter plate and free phosphate generated by the PP2A substrate dephosphorylation was detected by addition of the Malachite Green Phosphate Detection Solution and by measuring absorbance at 650 nm.

### Sphingosine kinase assay

SphK1 activity was measured with the sphingosine kinase activity assay kit (Echelon Biosciences, Salt Lake City, UT) according to the manufacturer instructions. MM cells where treated with 6 µM FTY720 or vehicle for 24 h and cell lysates were collected for the assay. ATP consumption due to SphK1 substrate phosphorylation was measured after 1.5 h of enzymatic reaction. Addition of 0.5% Triton to the lysis buffer ensured inhibition of SphK2 activity [42].

### Immunoblotting

Total cell protein extracts were prepared using *M*-*PER*™ Mammalian Protein Extraction Reagent (ThermoFisher, Waltham, MA) and quantified with Bio-Rad Protein Assay (Bio-Rad, Hercules, CA). The proteins were separated on a 4–12% gradient polyacrylamide gel, transferred to PVDF membranes (EMD Millipore, Billerica, MA) and probed with antibodies overnight. Pierce™ ECL western blotting substrate was used for detection. Densitometry analysis was conducted with Image J software (NIH, Bethesda, MD). The antibodies used were: caspase-3, PARP, pERK Tyr^204^, pAKT Ser^473^, total AKT, Lamin, VDAC (Cell Signaling Technologies, Danvers, MA), Bcl-2, ERK1/2, α-tubulin (Santa Cruz Biotechnology, Dallas, TX), PP2A_C_ clone 1D6 and GAPDH (EMD Millipore).

### Isolation of cytosol, mitochondria and nucleus

For the separation of mitochondrial and cytoplasmic fractions cells were collected in homogenization buffer (20 mM HEPES–KOH pH 7.5, 10 mM KCl, 1.5 mM MgCl2, 1 mM bisodium EDTA, 1 mM EGTA, sucrose) and gently disrupted by 22-gauge needle aspiration. Homogenates were centrifuged at 1000×*g* for 5 min at 4 °C to separate nuclei and unbroken cells, then supernatants were centrifuged at 10,000×*g* for 20 min in 4 °C to recover mitochondria. The pellets containing mitochondria were lysed with radioimmunoprecipitation assay buffer (RIPA) and the supernatants containing the cytoplasmic fraction were concentrated using Amicon Ultra-4 Centrifugal Filter (EMD Millipore).

For the separation of nuclear and cytoplasmic fractions, cell pellets were re-suspended in 400 μl of hypotonic buffer (10 mM Hepes pH 7.9, 10 mM KCl, 0.1 mM EDTA, 0.1 mM EGTA, 1 mM DTT and 0.5 mM PMSF) and incubated on ice for 15 min, to allow cell swelling. 25 μl of 10% NP-40 was added and samples were homogenized and the homogenates were centrifuged for 30 s at 14,000×*g* in 4 °C. Supernatants (cytosolic fraction) were collected and nuclear pellets were washed with PBS and centrifuged to remove cytoplasmic contamination. Nuclei were finally resuspended in hypotonic buffer (20 mM HEPES pH 7.4, 400 mM NaCl, 1 mM EDTA, 1 mM EGTA, 1 mM DTT, 1 mM PMSF), incubated on ice for 30 min, vortexed every 5 min, and then centrifuged at 16,000×*g* for 10 min. Supernatants were collected as nuclear homogenates.

### Balb/c mouse syngeneic MM model

Eight-week-old Balb/c mice (Taconic Biosciences, Hudson, NY), maintained in accordance with guidelines of the University of Hawaii Institutional Animal Care and Use Committee, were injected subcutaneously (s.c.) with AB1 cells (10^5^ cells/mouse). Tumors were detected on day 7 and mice were randomized into 2 groups (n = 15) to receive: FTY720 10 mg/kg or vehicle (2% DMSO solution in water) intraperitoneally (i.p.) in a 5-day-on, 2-day-off schedule. This FTY720 dosage is largely used in mouse cancer models (e.g. [23]), and was chosen according to safety and toxicity data disclosed by Novartis. Tumor volume (cm^3^) was measured using the formula: π/6 × larger diameter × (smaller diameter)^2^, as previously reported [43].

## Results

### FTY720 selectively suppresses MM cell viability and anchorage–independent growth without affecting normal mesothelial cells

**Fig. 1:**
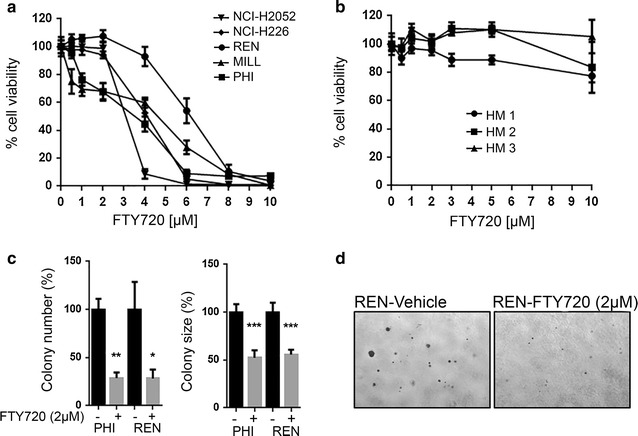
FTY720 suppresses viability of MM cells without significant effect on HM cells. **a**, **b** Alamar Blue viability assay was performed after 48 h of treatment with increasing concentrations of FTY720 on five MM cell lines and HM cells derived from three non-cancer patients. **c** Anchorage-independent growth was evaluated in REN and PHI cells upon treatment with 2 µM FTY720 or vehicle. The *graphs* represent the average number (*left*) and size of colonies (*right*) in three independent experiments, expressed as the percentage of vehicle. **d** Pictures representative of FTY720- or vehicle-treated REN cells are shown (original magnification 40×)

We assessed cell viability after 48 h treatment with increasing concentrations of FTY720 (0.5–10 μM) in a panel of human MM cells and in three HM cultures. FTY720 caused a significant decrease in cell viability of all MM cells tested in a dose-dependent manner (Fig. 1a), while no significant changes of viability were observed in HM (Fig. 1b).

To further verify the potential anti-cancer effect of FTY720 in MM, we performed soft agar colony formation assay on MM cells exposed to 2 µM FTY720 or vehicle for 4–6 weeks. The number of colonies that developed in FTY720-treated cells was significantly lower compared to cells that received only vehicle (*P* = 0.0145 for REN cells; *P* = 0.0006 for PHI cells). Similarly, the size of FTY720-treated colonies was also significantly smaller compared to those that developed in cells treated with vehicle (*P* < 0.0001 for both cell lines) (Fig. 1c, d).

FTY720 inhibits migration and invasion of some cancer cell types [26, 33, 34]. Therefore, we investigated whether FTY720 treatment would alter the migratory properties of MM cells. Three-hour treatment with 2 μM FTY720 did not alter migration of MM MILL and PHI cells (Additional file 1: Figure S1).

As some FTY720 functions, including its ability to modulate cell migration, have been ascribed to its phosphorylated metabolite, FTY720-P, we also treated MM cells for 48 h with increasing concentrations of FTY720-P. FTY720-P compound was biologically active, as indicated by its ability to induce phosphorylation of ERK in both MM and HUVEC cells in our control assay (Additional file 2: Figure S2A) [44]. However, treatment with FTY720-P did not suppress MM cell viability (Additional file 2: Figure S2B).

These results suggested that the non-phosphorylated form of FTY720 is effective, and selective, in reducing MM growth in vitro, at the same concentrations as observed in other cancer cells [45].

### FTY720 reactivates the tumor suppressor PP2A in MM

The tumor suppressor protein phosphatase PP2A and the pro-survival kinase SphK1, are key targets of FTY720 [32, 46]. We investigated whether these proteins are deregulated in MM and whether the observed anti-cancer effects of FTY720 in MM are mediated by these proteins.

**Fig. 2:**
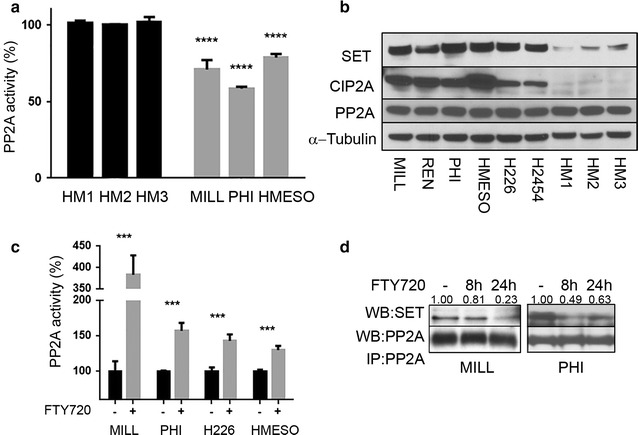
PP2A is inhibited in MM and FTY720 reactivates it. **a** Quantification of PP2A activity in indicated MM and HM cells was performed using a PP2A-specific enzymatic activity assay. Results are expressed as percentage of PP2A activity, as compared to PP2A activity detected in HM cells. Mean and SD of 3 replicates of a representative experiment, out of three performed, are given. **b** Representative western blot analysis of PP2A, SET and CIP2A protein levels in 6 human MM cell lines and 3 HM cultures. Levels of α-tubulin were detected as loading control. **c** Quantification of PP2A activity in indicated MM cells 24 h after treatment with 6 µM FTY720 or vehicle. Results are expressed as percentage of PP2A activity normalized to vehicle control, for each cell line separately. Means and SD of 3 replicates of a representative experiment, out of three performed, are given. **d** PP2A was immunoprecipitated from MILL and PHI cell cultures untreated or treated with FTY720 (6 µM) or vehicle for 8 and 24 h. Immunoprecipitates were probed with anti-SET antibody (*upper panel*) and anti-PP2A antibody, as loading control (*lower panel*). Relative densitometric analysis data, for SET protein levels, normalized to PP2A protein levels, are shown

In order to study the possible effects of FTY720 on PP2A in MM, we first investigated the status of PP2A and measured the activity levels of PP2A in three MM cell lines. We found that the PP2A activity was significantly lower in MM cells, as compared to HM cells (Fig. 2a), suggesting that the tumor suppressor functions of PP2A are impaired in MM. However, no differences were detected in the protein levels of PP2A between MM cells and HM (Fig. 2b). The nuclear oncogene SET and cancerous inhibitor of protein phosphatase 2A (CIP2A) are endogenous inhibitors of PP2A activity [47]. Therefore, we investigated the levels of SET and CIP2A in a panel of MM cells and HM by immunoblotting. We found that all six MM cell lines tested showed significant elevation in SET and CIP2A protein levels compared to HM (Fig. 2b). These data suggested that PP2A deregulation by its inhibitors may contribute to the malignant phenotype of MM. Because PP2A is a target of FTY720, we investigated whether FTY720 may influence PP2A activity in MM cells. We found that, upon treatment with FTY720, PP2A phosphatase activity was significantly increased (Fig. 2c), indicating that FTY720 may reactivate the tumor suppressor functions of PP2A in MM. It has been reported that SET directly binds and inhibits PP2A [48], and that FTY720 reactivates PP2A via displacement of SET from the PP2A complex [24, 46]. We performed immunoprecipitation of PP2A and detected SET in the immune-complexe from MM cells treated with FTY720 or vehicle. Our results indicated that FTY720 treatment resulted in displacement of SET from the PP2A complex in MM (Fig. 2d). Moreover, SET protein expression levels were not modulated upon FTY720 treatment (Additional file 3: Figure S3), indicating that the lower levels of SET found in the immunoprecipitation resulted from decreased binding of SET to PP2A. CIP2A levels were also unchanged, indicating that FTY720 does not modulate the expression levels of these PP2A inhibitors in MM (Additional file 3: Figure S3).

To further investigate the anti-cancer effect of FTY720 in MM, we examined SphK1, another target of FTY720 that was shown to be overexpressed in MM [35]. We found that FTY720 significantly reduced SphK1 kinase activity in MM cells (Additional file 4: Figure S4).

Altogether, these data indicated that FTY720 is effective in inhibiting MM growth and this mechanism is, at least in part, mediated by re-activation of PP2A tumor suppression function via SET inhibitor displacement and suppression of SphK1 activity.

### FTY720 induces programmed cell death in MM cells

Both SphK1 and PP2A have pleiotropic activities and modulate a large number of downstream effectors. Among these, a critical effector regulated by both SphK1 and PP2A is the AKT kinase [49, 50]. AKT is involved in the balance between cell survival and death via its interaction with members of the Bcl-2 family [51, 52].

**Fig. 3:**
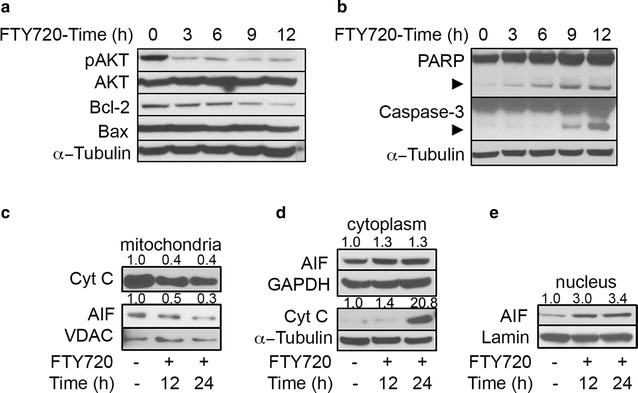
FTY720 induces programmed cell death in MM cells. **a** MILL cells were treated with 6 µM FTY720 and the levels of phosphorylated AKT (Ser^473^), total Bcl-2 and Bax proteins were assessed by immunoblot analysis at indicated time-points. Levels of α-tubulin were detected as loading control. **b** Activation of PARP and caspase-3 was evaluated in REN cells by immunoblot analysis after treatment with 6 µM FTY720 at indicated time points. *Arrows* indicate caspase-3 and PARP cleaved forms. **c**–**e** Cyt-c and AIF release in PHI cells were assessed by immunoblot analysis. Cells were treated with 6 µM FTY720 or vehicle for 12 and 24 h. Fractionation was performed as described in the “Methods” section. VDAC, lamin and α-tubulin were used as controls of loading and fractions’ purity. Numbers above blotting *figures* indicate the band densitometric analysis data, relative to loading control. Figures show results from one experiment representative of two independently performed. All immunoblot experiments were repeated with two other MM cell lines with comparable results

Notably, upon treatment with FTY720, levels of phosphorylated (Ser^473^) AKT gradually decreased in a time–dependent manner, while total AKT levels remained constant, suggesting inactivation of this kinase (Fig. 3a), Bcl-2 protein levels decreased in a time–dependent manner, with a longer kinetics starting 9 h after FTY720 treatment, while the levels of the pro-apoptotic protein Bax remained stable (Fig. 3a).

The reduction of AKT phosphorylation associated with the reduction of Bcl-2 levels as well as the high Bax/Bcl-2 ratio suggested that FTY720 could induce apoptosis in MM cells. The levels of PARP and caspase–3 cleaved forms increased upon FTY720 treatment, in time- and dose–dependent manner (Fig. 3b; Additional file 5: Figure S5A). We then performed annexin V (AV) and propidium iodide (PI) staining. Upon FTY720 treatment, the percentage of both early apoptotic cells and late apoptotic cells increased (average increase of about 9 and 11% respectively) (Additional file 5: Figure S5B). Moreover, FTY720 caused a progressive decrease of the proteins cytochrome C (Cyt-c) and the apoptosis inducing factor (AIF) in the mitochondrial fraction (Fig. 3c) with subsequent increase of Cyt-c and AIF in the cytoplasm (Fig. 3d), and of AIF in the nuclear fraction (Fig. 3e).

Altogether, our data indicate that FTY720 treatment is associated with Bcl-2 degradation, activation of PARP and effector caspase 3 and with translocation from the mitochondria of Cyt-c and AIF, known events of the caspase–dependent and possibly also caspase–independent programmed cell death pathways.

### FTY720 is an effective inhibitor of tumor growth in a MM syngeneic mouse model

In parallel, we investigated the anti-cancer potential of FTY720 in vivo. Since FTY720 is an immunomodulatory agent, we evaluated its efficacy and toxicity using a syngeneic mouse MM model. Balb/c mice bearing subcutaneous AB1 [53] tumors of 5 mm in diameter were randomized to receive treatment with 10 mg/kg dose of FTY720 or vehicle.

**Fig. 4:**
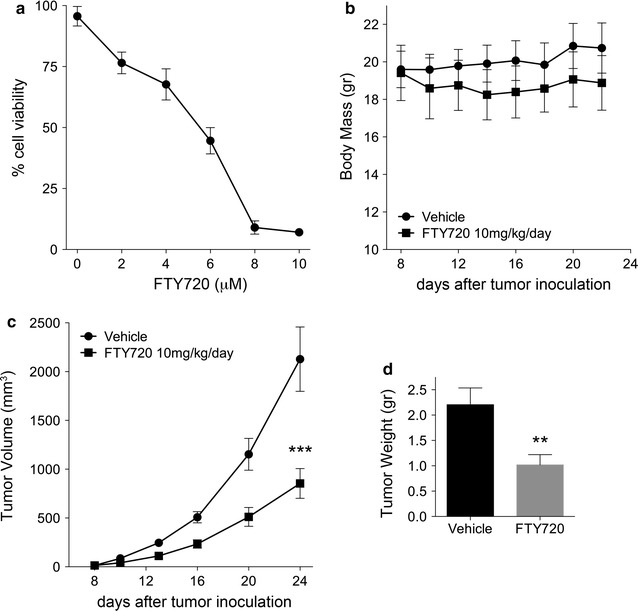
FTY720 reduces tumor growth in MM syngeneic model without causing toxicity. **a** Suppression of viability of AB1 mouse cell line in vitro upon 48 h of treatment with indicated FTY720 doses. **b**–**d** Balb/c mice inoculated subcutaneously with AB1 mouse cells were treated with 10 mg/kg FTY720 or vehicle (n = 15 mice per group) on a 5/2 on/off schedule. Treatment was initiated once tumors reached a palpable size of 0.5 mm. Mice were weighted throughout the duration of the study to monitor for system toxicity **b**. Tumor volumes were measured every 3 days and growth curve was plotted **c**. Final tumor weights were also measured **d**

The response of AB1 mouse cells to FTY720 was first tested in vitro by Alamar Blue assay. FTY720 effectively reduced viability of AB1 cells with GI_50_ = 5 µM (Fig. 4a). Signaling downstream of FTY720 treatment in AB1 was also assessed (Additional file 6: Figure S6). We found that FTY720 induces similar effects in AB1 mouse MM cells, as compared to human MM cells, including SET displacement from PP2A complex (Additional file 6: Figure S6A), reduction of p-AKT (Ser^473^) and Bcl-2, and induction of caspase-3 cleavage (Additional file 6: Figure S6B).

In mice, fifteen doses of FTY720 were administered by i.p. injection on a 5-days-on, 2-days-off schedule. Upon this treatment regimen, we did not observe significant weight loss (Fig. 4b), or changes in grooming, posture, hydration, stools and social behaviors. Moreover, no spleen size enlargement, or discoloration or any other morphological abnormalities of internal organs were observed, indicating that the drug was well tolerated in immune-competent mice at the dosage applied.

Tumor size was measured every 3 days and all mice were sacrificed on day 24, when tumors in the control group reached the limit of 2 cm in size, as per our University of Hawaii IACUC regulations. Tumor weight was determined immediately after tumor removal. FTY720, at 10 mg/kg dose, significantly inhibited tumor volume by 60% average at treatment end-point (*P* = 0.0009) (Fig. 4c). The average tumor weight was also significantly lower in FTY720–treated mice (*P* = 0.0065) (Fig. 4d).

Altogether, our data in vivo demonstrated that FTY720 is effective in reducing MM tumor growth without causing significant toxicity, supporting its potential as an effective anti-cancer agent in MM.

## Discussion

MM has a dismal prognosis, with a median survival of about 1 year. The current mainstays of MM therapy are represented by surgery—mostly palliative, and platinum-based chemotherapy combined with pemetrexed [54]. Recently, the addition of the angiogenesis inhibitor bevacizumab resulted in a marginal increase in overall survival [55]. Novel therapeutic agents are therefore needed to tackle this deadly disease.

The development of new therapeutic agents for rare cancers, such as MM, is hampered by the increasing costs of research and drug development from the laboratory to the patient’s bed [56]. Drug repurposing, which involves finding new uses for existing drugs that are outside the scope of their original indication, is a strategy that drastically reduces time and costs to bring a new drug to the market.

The anti-neoplastic properties of FTY720 are well established and evidences for repurposing this agent have been recently proposed [22]. Effects of FTY720 as a single agent or in combination with chemotherapy have been shown in a wide variety of cancer models, such as leukemia, colorectal, lung, brain and breast cancers [22]. So far, the efficacy and mode of action of FTY720 in MM, has not been thoroughly investigated. In this study we show that FTY720 effectively reduced MM growth both in vitro and in vivo as a single agent. Our data indicate that, in MM, FTY720 significantly inhibited the enzymatic activity of the oncogene SphK1 and reactivated the tumor suppressor activity of PP2A, suggesting that FTY720 anti-tumor activity may be exerted via modulation of these effectors. SphK1 has been previously reported to be upregulated in MM [35]. Here we show that MM cells exhibit PP2A inactivation, associated with overexpression of PP2A inhibitory proteins SET and CIP2A, indicating that PP2A may be involved in MM malignancy. Indeed, animal studies have shown that inactivation of PP2A by the SV40 small-t tumor antigen is required for SV40-mediated transformation of primary human mesothelial cells in tissue culture and for SV40-induced MMs in hamsters [57–59]. A number of studies indicate that alterations of PP2A and SphK1 (namely, inhibition of the former and overexpression of the latter) cooperate in the activation of pro-survival pathways in cancer [60–62]. Here we show that FTY720 caused inhibition of AKT phosphorylation and reduced the levels of anti-apoptotic Bcl-2 protein, leading to programmed cell death. Overall our mechanistic data provide a solid rationale for the therapeutic efficacy of FTY720 observed in MM.

Since the discovery of its potent anticancer activity, repurposing of FTY720 is being considered for cancer patient treatment [22, 56]. Efficacy and bio-safety profiles of FTY720 are well described in multiple sclerosis (MS) patients [63]. In cancer therapy applications, its possible undesired effects and the interference of its immunosuppressive activities are recognized [22, 56]. Toxicity and efficacy trade-offs are not straightforward in cancer treatment: the actual toxicity associated with the use of a drug depends on many factors including the duration and treatment regimen, as well as patient characteristics, such as tumor staging, age and other co-morbidity factors. However, in highly aggressive and resistant cancers, such as MM, the survival benefits obtained administering an effective anticancer drug, may outweigh the risks of toxicity. A recent meta-analysis study of safety and toxicity of FTY720, designed to investigate its potential use in the treatment of chronic myeloid leukemia, suggested that this drug has an acceptable risk to benefit ratio, given its lack of bone marrow toxicity and relatively low rate of serious side effects [64]. In future, biomarker studies aimed to identify those patients that may be more likely to benefit from FTY720 therapy, would help target this drug to a selected patient subpopulation, where benefits outweigh risks [65–67]. To note, the side effects of FTY720 are reversible upon treatment withdrawal, at least at the doses used in MS patients [68], therefore, a design of a specific on/off schedule may mitigate the adverse effects. Moreover, a number of different strategies are being developed to minimize FTY720 side effects, such as nanoparticle delivery, combination therapies and design of new derivatives of FTY720. Most importantly, the adverse effects of FTY720 are attributed to its phosphorylated isoform, FTY720-P, while the anti-cancer effects, exerted via interaction with targets, such as SET-PP2A and SphK1, have been specifically associated with the unphosphorylated form of FTY720 [32, 46]. Accordingly, unphosphorylatable second-generation derivatives of FTY720 have been developed and are being extensively studied in preclinical cancer models (e.g. OSU-2S0 [69]). As compared to FTY720, these appear to possess a stronger anti-cancer effect, while lacking systemic immunosuppressive properties [69]. Such derivatives should eliminate concerns related to immune suppression and represent a novel promising therapeutic strategy for MM.

## Conclusions

Our data represent a proof of principle for the efficacy of FTY720 in MM therapy, with no apparent toxicity in our mouse model. FTY720 and its second-generation derivatives, potentially fit the criteria for drug repurposing and can be promising anticancer agents for the treatment of MM, most likely, in combination with existing or novel therapies.

## Additional file

**Additional file 1: Figure S1.** FTY720 does not affect MM cell migration.

**Additional file 2: Figure S2.** FTY720-P does not affect MM cell survival.

**Additional file 3: Figure S3.** FTY720 does not alter SET and CIP2A protein expression levels in MM cells.

**Additional file 4: Figure S4.** FTY720 inhibits SphK1.

**Additional file 5: Figure S5.** FTY720 induces apoptosis in MM cells.

**Additional file 6: Figure S6.** FTY720 displaces PP2A inhibitor protein –SET, reduces pAKT and Bcl2, and activates apoptosis in AB1 mouse cells.

## Abbreviations

AKT: serine/threonine kinase 1
Bcl-2: B-cell CLL/lymphoma 2
CIP2A: cancerous inhibitor of protein phosphatase 2A
EDTA: ethylenediaminetetraacetic acid
EGTA: ethylene glycol-bis(β-aminoethyl ether)-N,N,N’,N’-tetraacetic acid
ERK1/2: extracellular signal-regulated kinases ½
FBS: fetal bovine serum
FITC: fluorescein isothiocyanate
HM: human mesothelial cells
Hr: hours
MM: malignant mesothelioma
SphK1: sphingosine kinase 1
PARP: poly(ADP-Ribose) polymerase
PI: propidium iodide
PS: phosphatidylserine
PP2A: protein phosphatase 2A
PVDF: polyvinylidenefluoride
TRIS: trisaminomethane
WT-1: Wilms tumor 1
VDAC: voltage-dependent anion channel
