# Supplementary material for: FTY720 inhibits mesothelioma growth in vitro and in a syngeneic mouse model
Source: J Transl Med. 2017 Mar 15;15:58. doi: 10.1186/s12967-017-1158-z (PMC5353897; doi:10.1186/s12967-017-1158-z)
Supplement: Supplementary file 1 — Additional file 1: Figure S1. FTY720 does not affect MM cell migration. [file 12967_2017_1158_MOESM1_ESM.pdf]

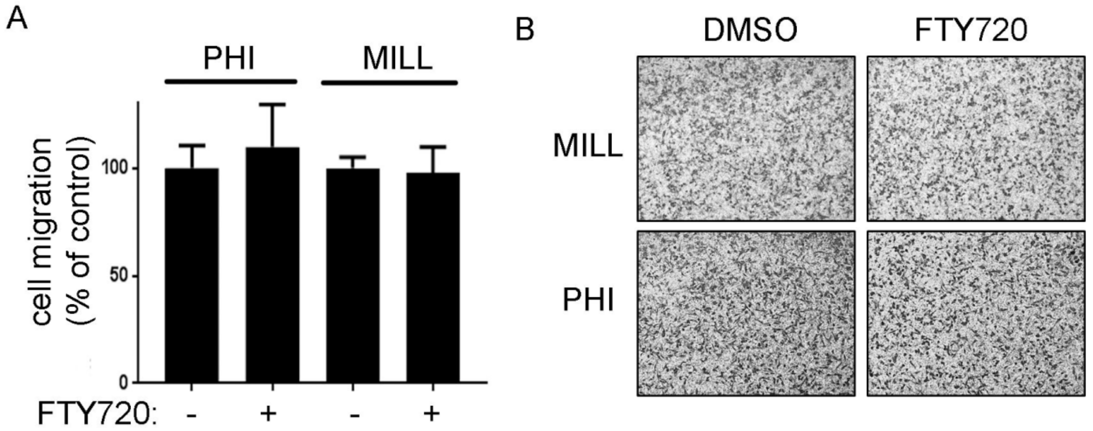

Figure S1. FTY720 does not affect MM cell migration. (A) MILL and PHI cells were treated with FTY720 (2 μM) or DMSO and subjected to the migration assay. Graph shows mean percentages of the migrated cells in treated vs. untreated samples, counted from two independent experiments. **(B)** Representative photomicrographs showing HEMA 3 staining of MILL and PHI cells migrated and adhered to the lower surface of insert membrane (original magnification 100x).
