# Supplementary material for: FTY720 inhibits mesothelioma growth in vitro and in a syngeneic mouse model
Source: J Transl Med. 2017 Mar 15;15:58. doi: 10.1186/s12967-017-1158-z (PMC5353897; doi:10.1186/s12967-017-1158-z)
Supplement: Supplementary file 2 — Additional file 2: Figure S2. FTY720-P does not affect MM cell survival. [file 12967_2017_1158_MOESM2_ESM.pdf]

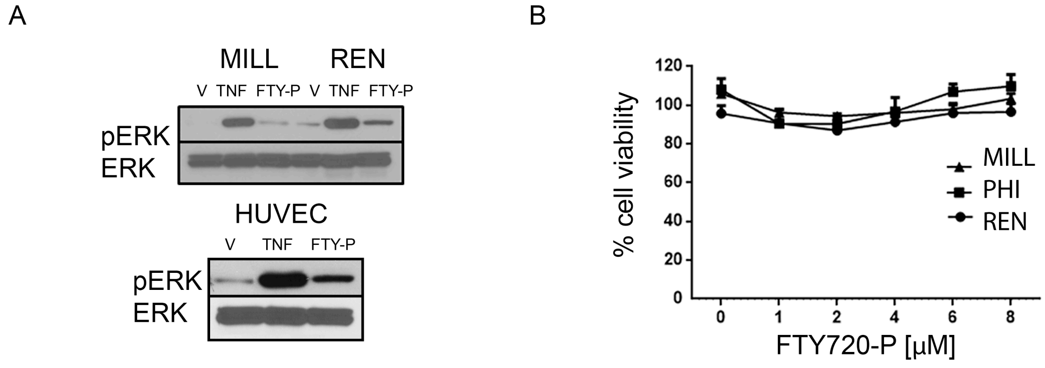

Figure S2. FTY720-P does not affect MM cell survival. (A) Phosphorylation of ERK1/2 in REN, MILL and HUVEC after stimulation with 1 µM FTY720-P (5 min) was evaluated by immunoblot analysis, as a positive control of drug activity. TNFα (1 ng/ml, 30 min) stimulation was used as a positive control for ERK phosphorylation; total ERK protein levels were detected as loading control. **(B)** MILL, REN and PHI cells were treated with increasing concentrations of phosphorylated FTY720 (FTY720-P) and cell viability was assessed by Alamar Blue assay, upon 48h treatment.
