# Supplementary material for: FTY720 inhibits mesothelioma growth in vitro and in a syngeneic mouse model
Source: J Transl Med. 2017 Mar 15;15:58. doi: 10.1186/s12967-017-1158-z (PMC5353897; doi:10.1186/s12967-017-1158-z)
Supplement: Supplementary file 3 — Additional file 3: Figure S3. FTY720 does not alter SET and CIP2A protein expression levels in MM cells. [file 12967_2017_1158_MOESM3_ESM.pdf]

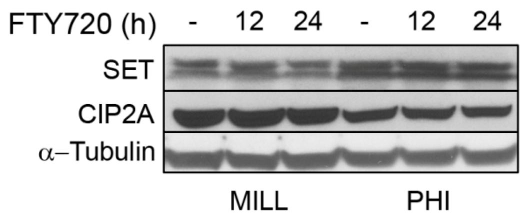

Figure S3. FTY720 does not alter SET and CIP2A protein expression levels in MM cells. Representative western blot analysis of SET and CIP2A protein expression levels in MILL and PHI cells, upon 12 hr and 24 hr treatment with 6 μM FTY720.
