# Supplementary material for: FTY720 inhibits mesothelioma growth in vitro and in a syngeneic mouse model
Source: J Transl Med. 2017 Mar 15;15:58. doi: 10.1186/s12967-017-1158-z (PMC5353897; doi:10.1186/s12967-017-1158-z)
Supplement: Supplementary file 4 — Additional file 4: Figure S4. FTY720 inhibits SphK1. [file 12967_2017_1158_MOESM4_ESM.pdf]

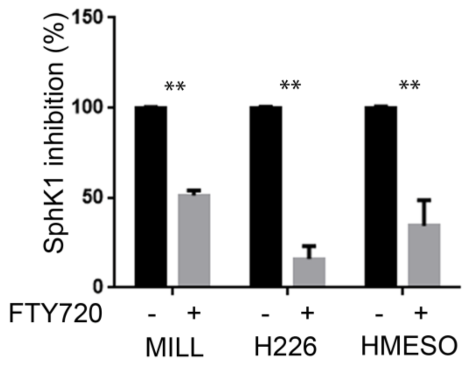

Figure S4. FTY720 inhibits SphK1. Quantification of SphK1 activity in indicated MM cells was performed using SphK1 enzymatic activity assay, 24 hr after treatment with 6 μM FTY720 or vehicle. Results are expressed as percentage of SphK1 activity, normalized to vehicle control, for each cell line separately. Mean and SD of 3 replicates of a representative experiment, out of three performed, are given.
