# Supplementary material for: FTY720 inhibits mesothelioma growth in vitro and in a syngeneic mouse model
Source: J Transl Med. 2017 Mar 15;15:58. doi: 10.1186/s12967-017-1158-z (PMC5353897; doi:10.1186/s12967-017-1158-z)
Supplement: Supplementary file 5 — Additional file 5: Figure S5. FTY720 induces apoptosis in MM cells. [file 12967_2017_1158_MOESM5_ESM.pdf]

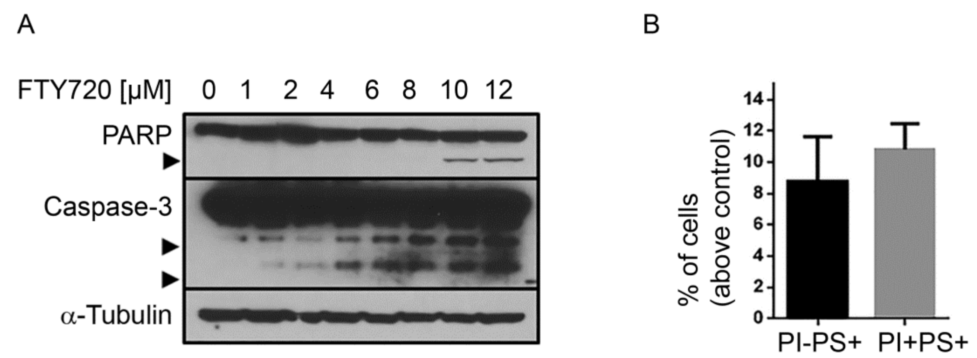

Figure S5. FTY720 induces apoptosis in MM cells. (A) Dose-dependent effect of FTY720 on caspase-3 and PARP cleavage in MILL cells upon 12 hr of treatment was assessed by immunoblot analysis. Arrowheads indicate cleaved caspase-3 and PARP fragments. α-tubulin was detected as loading control. **(B)** Flow cytometry analysis of Annexin V positive, PI negative (PI-PS+) and Annexin V and PI positive (PI+PS+) REN cells after 16 hr of treatment with 6 μM FTY720. Bars represent mean and SD of percentage differences between cell counts for FTY720-treated and untreated samples.
