# Supplementary material for: FTY720 inhibits mesothelioma growth in vitro and in a syngeneic mouse model
Source: J Transl Med. 2017 Mar 15;15:58. doi: 10.1186/s12967-017-1158-z (PMC5353897; doi:10.1186/s12967-017-1158-z)
Supplement: Supplementary file 6 — Additional file 6: Figure S6. FTY720 displaces PP2A inhibitor protein –SET, reduces pAKT and Bcl2, and activates apoptosis in AB1 mouse cells. [file 12967_2017_1158_MOESM6_ESM.pdf]

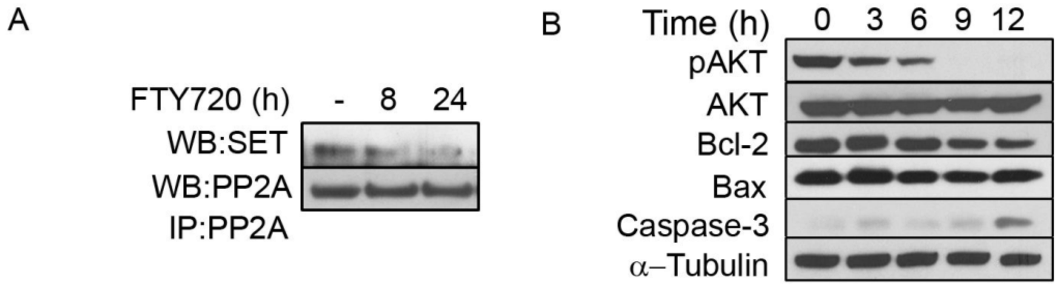

Figure S6. FTY720 displaces PP2A inhibitor protein –SET, reduces pAKT and Bcl2, and activates apoptosis in AB1 mouse cells. (A) PP2A was immunoprecipitated from AB1 cell cultures untreated or treated with vehicle or 6 μM FTY720 for 8 hr and 24 hr. Immunoprecipitates were probed with anti-SET antibody (upper panel) and anti-PP2A antibody, as a loading control (lower panel). **(B)** AB1 cells were treated with vehicle or 6 μM FTY720 for 3, 6, 9 or 12 hr and the levels of phosphorylated AKT (Ser^{473}), AKT, total Bcl-2, Bax and cleaved caspase-3 proteins were assessed by immunoblot analysis at indicated time-points. Levels of α-tubulin were detected as loading control.
